# Perceived neighbourhood social cohesion and depressive symptom trajectories in older adults: a 12-year prospective cohort study

**DOI:** 10.1007/s00127-018-1548-4

**Published:** 2018-06-19

**Authors:** Milagros Ruiz, Shaun Scholes, Martin Bobak

**Affiliations:** Research Department of Epidemiology and Public Health, University College London, 1-19 Torrington Place, London, WC1E 6BT UK

**Keywords:** CES-D, Depression, Latent growth modelling, Neighbourhood, Social cohesion, Trajectories

## Abstract

**Purpose:**

To model the dynamic age-related rate of change in depressive symptomatology in later life and to test the hypothesis that low perceived neighbourhood social cohesion is associated with steeper trajectories of depressive symptoms in older adults.

**Methods:**

We analysed data on 11,037 participants aged 50+ from the English Longitudinal Study of Ageing. Perceived social cohesion (PSC) of participants’ neighbourhoods was assessed at baseline (2002/2003). Depressive symptoms were measured using CES-D scores (ranging from 0 to 8) on 7 occasions from baseline to 2014/2015. Trajectories of depressive symptoms by baseline PSC were estimated using latent growth modelling.

**Results:**

At baseline, adults with low PSC had more depressive symptoms than age counterparts with high PSC. Consistent with the U-shaped trajectory of depressive symptoms by age, the association between PSC tertile and changes in depressive symptoms over follow-up was modified by age. Fifty-year-old participants with low PSC reported an average decrease in CES-D score from 0.66 to 0.54 during the 12-year follow up, compared to a change from 0.47 to 0.34 for age counterparts with high PSC. By contrast, in persons aged 85 at baseline, the mean CES-D score increased from 1.09 to 1.30 for participants with high PSC, while the rise was greater (from 1.49 to 2.03) among those with low PSC. The main effects and interaction of PSC with age were robust to adjustment for socio-economic and health characteristics.

**Conclusions:**

Depressive symptom trajectories by PSC appear to widen as adults reach old age.

**Electronic supplementary material:**

The online version of this article (10.1007/s00127-018-1548-4) contains supplementary material, which is available to authorized users.

## Introduction

Depression is an increasingly important disorder with significant social and public health consequences [1, 2]. It has been estimated that in England depression affects 20% of adults aged 65–69 years and this proportion is doubled in adults aged 85 and over [3]. This burden of depression at older ages is growing rapidly, as older persons make up the fastest growing age group of the total population [4]. Among older persons, say over 60–65 years old, the prevalence of depression increases with age [5]. But over the wider life course, depression follows a U-shaped trajectory from middle to older ages; as the prevalence declines during early middle ages, plateaus at middle age and increases again at older ages [6, 7].

The aetiology of depression in later life includes multiple risk factors that influence older adults’ ability to confront changing life circumstances, stressful events and declining states of physical health [1, 8]. Among these factors, neighbourhood social cohesion, measured at both the individual- and community-level [9, 10], has been linked with the risk of depression in older adults [11–18]. Social cohesion has been defined as a component of cognitive/perceptual social capital that consists of altruism, reciprocity, values and norms that are shared among neighbours [10, 12]. Neighbourhood social cohesion may function as a buffer on individual-level stressors [13], and increase residents’ access to community-level resources that lessen the risk of depressive disorders [19]. Among older adults, the neighbourhood context is conceivably more important, due to age-based limitations and community-level structural barriers that reduce their capacity to manage environmental stressors [17, 18]. As adults become increasingly vulnerable to their neighbourhood context in later life and thus require more care, social participation and support become irrevocably dwindled as a result of death of family and friends and reduced social networks [18]. Other life course transitions, such as retirement, are also linked to profound changes in social, travel and role activities [20]; which may increase the time spent within the local area and reduce the opportunity for social interaction outside one’s neighbourhood [19, 21].

Although neighbourhood social cohesion may be protective against depression during the later years, most research on older adults is cross-sectional, and therefore, cannot assess how this association varies amidst cohort ageing [12, 13, 17, 18]. Noteworthy longitudinal studies have confirmed a prospective relationship, but the limited follow-up period (1–3 years) preclude the assessment of trajectories of ageing [11, 16]. Very few studies have assessed the interplay between the effects of ageing and neighbourhood social cohesion on the rate of change in depressive symptoms during this particular stage of life, with inconsistent results [14, 15]. Therefore, it is unclear whether the protective effect of neighbourhood social cohesion on depressive symptomatology varies systematically with age.

The present study investigated whether depressive symptom trajectories differ by perceived neighbourhood social cohesion among middle-aged and older English adults over a 12-year period, and assesses whether the longitudinal association differs by age. As part of these analyses, we also examined whether the (hypothesised) longitudinal association is confounded or modified by socio-economic and physical health characteristics.

## Methods

### Study population

The English Longitudinal Study of Ageing (ELSA) is a population-based prospective cohort study designed to monitor the long-term health of older adults (aged 50+ years) living in private households throughout England. Baseline assessment of the original core sample (*n* = 11,391) took place in 2002/2003 and yielded an individual 67% response rate [22]. Follow-up assessments at two-yearly intervals have been conducted up to 2014/2015, whereby 43.0% (*n* = 4894) of the original core sample participated at wave 7 [23]. The present study uses data collected from the core sample at waves 1–7, which provided prospective data on depressive symptoms ascertained over a 12-year period.

### Measures

Depressive symptomatology was assessed using the eight-item version of the Center for Epidemiological Depression (CES-D) scale at each wave. The CES-D scale is a well-validated screening instrument that identifies those at risk of depressive disorders in the general population, including older adults [24, 25]. The 8-item version was demonstrated to measure depressive symptomatology with a precision comparable to the original 20-item scale [26]. The CES-D 8 asks whether: three symptoms on depressed affect (‘I felt depressed,’ ‘I felt lonely,’ ‘I felt sad’); three symptoms on somatic and retarded activity (‘I felt that everything I did was an effort,’ ‘My sleep was restless,’ ‘I could not get “going”’); and two symptoms on positive affect (‘I was happy,’ ‘I enjoyed life’); were experienced for ‘much of the time during the past week’ [27, 28]. Participants provided yes/no responses to each question. Affirmative and negative responses to the first six and last two questions, respectively, were coded as 1. Item responses were summed to generate a count score ranging from 0 to 8 for participants who answered at least six of the eight questions at each wave, as scoring guidelines require complete data on at least 75% of the CES-D scale [27].

Perceived neighbourhood social cohesion (PSC) was measured by the grid question, ‘How do you feel about your local area, that is everywhere within a 20 min walk or about a mile from your home?’, at wave 1. Four items from the grid question were selected a priori as indicators of PSC: (1) ‘I really feel a part of this area’/‘I feel that I don’t belong in this area;’ (2) ‘Most people in this area can be trusted’/‘Most people in this area can’t be trusted;’ (3) ‘Most people in this area are friendly’/‘Most people in this area are unfriendly;’ and (4) ‘People in this area will take advantage of you’/‘People in this area will always treat you fairly.’ Participants answered on a bipolar Likert scale ranging from 1 to 7, denoting how closely they agreed to the opposing statements. Internal consistency of these items was deemed acceptable by Cronbach’s alpha (*α* = 0.73). Convergent validity of the construct was established by confirmatory factor analysis (*β* ≥ 0.55 for each item). The first three items were reverse coded for comparability; and item responses were summed to derive a score ranging from 4 to 28, which was rescaled from 0 to 24 for ease of interpretation. Higher scores indicate greater levels of PSC. As the distribution of PSC scores was negatively skewed, data were combined into high, medium and low tertiles.

Baseline covariates included continuous age, gender, white/non-white group and self-reported ever doctor diagnosis of depression—to account for prior depressive episodes; as well as socio-economic (educational qualification, economic activity, total non-pension wealth quintiles) and physical health (self-rated health and self-reported limiting long-term illness) indicators.

### Analytic sample

Of the 11,391 participants from the original core sample, 96.9% (*n* = 11,037) answered at least six of the eight CES-D items at wave 1. Counts of depressive symptoms were not derived for the remainder of the core sample because 2.1% (*n* = 241) did not complete this segment of the personal interview, and 0.6% (*n* = 67) responded to fewer than six of the CES-D items. A longitudinal dataset was obtained for these 11,037 participants, and included all repeat assessments for which 6+ CES-D items had been answered. Thirty-five percent (*n* = 3839) of the analytic sample had sufficient item response at all seven waves; and 51.7% (*n* = 5706) of participants had these data for at least five of the seven waves. The analytic sample had approximately 4.4 assessments of CES-D 8 data per participant (of a possible total of seven).

### Statistical analyses

Missing data were addressed by multiple imputation by chained equations which generated 20 imputed datasets. The imputation addressed item non-response to the CES-D among participants with 1 or 2 missing item responses across waves, to calculate count scores for the longitudinal analytic sample with sufficient data. Missing items were imputed using all available CES-D data collected at that particular wave, and all previous waves (if applicable) to improve the prospective reliability of imputed values. The CES-D data were included in the imputation model, but not imputed for participants who were missing 3+ CES-D items at any wave. The imputation also addressed missing data on PSC (11.8%, *n* = 1305) and covariates. As work status, self-rated health, and limiting long-term illness have been shown to predict longitudinal attrition in ELSA [29], their inclusion in the imputation and analysis model, respectively, aided to meet the assumption that data were missing at random.

Trajectories of change in the number of depressive symptoms over the study period were assessed using latent growth modelling [30]. Latent growth modelling is advantageous for the present analytic sample, as it incorporates participants with varying number of repeat assessments [31] under the assumption that data are missing at random using full information maximum likelihood estimation [32]. Individual growth processes are extracted from the repeat assessments of depressive symptoms across waves 1–7, and are conceptualised as continuous intercept and slope latent variables that denote baseline status (wave 1) and the rate of change (waves 2–7). The factor loadings of the intercept latent variable to all assessments were fixed to 1. The linear slope latent variable was specified with factor loadings to each assessment using years of follow-up divided by 12 as the time scale: 0 (wave 1), 0.17, 0.33, 0.50, 0.67, 0.83, and 1 (wave 7). Hence, the slope denotes the estimated total rate of change over the 12-year study period. Preliminary analyses (not described here) confirmed that a linear slope best fitted the longitudinal changes of the number of depressive symptoms in the analytic sample. The two latent factor model was fitted with a quadratic and cubic polynomial slope latent variable to assess non-linear rates of change, but the additional slope variables were not statistically significant. The latent growth models of the count data were estimated using negative binomial regression.

Initially, the intercept and linear slope were regressed on PSC and other baseline characteristics of age (centred at 65 years), gender, white/non-white group and self-reported ever doctor-diagnosis of depression. The initial model was tested for cohort and interaction effects, respectively. Baseline age squared was added to identify a cohort effect, independent of the age effect captured by the baseline age variable. As age squared was statistically significant on the intercept (*p* < 0.001) only, and not on the slope (*p* = 0.508); it was deemed that the significant age effect on the rate of change (*p* < 0.001) was not influenced by a cohort effect. Interaction terms between baseline age and PSC were statistically significant on both the baseline number of depressive symptoms and the rate of change, and therefore, retained in the model to account for widening age differences in depressive symptoms by PSC. Subsequently, the intercept and slope were further regressed on socio-economic and physical health characteristics at baseline. Initial and fully-adjusted model estimates were used to calculate and display depressive symptom trajectories by high and low PSC tertiles for 5-year age categories (ranging from 50 to 85) at baseline, hereafter referred to as 5-year cohorts.

### Sensitivity analyses

We addressed whether: (1) probable depressed cases could bias associations between self-reported PSC and self-reported depressive symptoms; and (2) PSC was associated not only with the number of depressive symptoms and rate of change over follow-up, but also with the onset of clinically relevant depressive symptomatology. Both analyses were restricted to participants with 0–2 depressive symptoms (below the risk threshold for the CES-D 8) [26] at baseline (*n* = 8269). First, we repeated the latent growth model with Poisson regression, as the baseline count data were no longer over-dispersed after removing counts from 3 to 8. Second, we fitted discrete time proportional hazards models to assess the incidence of probable depression (3 ≥ symptoms) by PSC tertiles over the 12-year period. Discrete time modelling considers that participants may report 3 ≥ symptoms more than once across waves; and that events were recorded at each wave, and not on a continuous time scale. The length of follow-up was the duration (months) from the baseline interview date to either: the date of the follow-up interview during which the participant first reported 3 ≥ symptoms; or during which censoring took place due to non-response. A Kaplan–Meier failure plot described the crude association between PSC and incident probable depression. Two discrete time proportional hazards models were estimated, which mirrored the nested modelling approach of the main analyses.

### Other methodological details

All analyses incorporated the non-response weight variable at wave 1 to ensure the analytic sample is representative of community dwelling adults aged 50+ at baseline; and were averaged over the multiply imputed datasets. Data imputation, descriptive and discrete time proportional hazard modelling were carried out using Stata V.13. Latent growth modelling was performed using Mplus V.7.

## Results

### Analytic sample characteristics

Table 1 reports the study characteristics of the longitudinal analytic sample. From 2002/2003 to 2014/2015, the mean number of depressive symptoms varied from 1.3 to 1.6. PSC scores of the high, medium and low population tertiles ranged from 22 to 24, 18–21 and 0–17, respectively. There was a slightly larger share of participants in the high PSC tertile (35%). Participants had a mean baseline age of 65 years, were 54% female and 5% had reported having been diagnosed with depression by a doctor.

**Table 1:** Study characteristics of the longitudinal analytic sample

Study data^a^		
Longitudinal measures	Mean	*n*
Number of depressive symptoms during the past week (0–8)		
Wave 1 (2002/2003)	1.6	11,037
Wave 2 (2004/2005)	1.6	8519
Wave 3 (2006/2007)	1.5	7248
Wave 4 (2008/2009)	1.4	6300
Wave 5 (2010/2011)	1.5	5843
Wave 6 (2012/2013)	1.3	5291
Wave 7 (2014/2015)	1.4	4571

Baseline measures	Mean or %	*n*
Perceived social cohesion score (0–24)		
High (22–24)	35.3	3896
Medium (18–21)	32.5	3587
Low (0–17)	32.2	3554
Baseline age	65.2	11,037
Female	53.7	5927
Non-white	2.7	298
Self-reported ever doctor-diagnosis of depression	5.4	596
No educational qualification	43.1	4757
Economic activity		
Employed	33.1	3653
Retired	49.9	5507
Economically inactive	7.4	817
Permanently sick/disabled	9.6	1060
Total non-pension wealth		
1 (wealthiest)	19.7	2174
2	19.8	2185
3	20.1	2218
4	20.2	2230
5	20.2	2230
Self-rated health		
Very good	20.0	2207
Good	33.9	3742
Fair	27.6	3046
Poor	18.5	2042
Self-reported limiting long-term illness	34.3	3786

^a^The estimates are averaged over the multiply imputed data sets, and corrected for the study’s non-response at wave 1. The longitudinal analytic sample (*n* = 11,037) includes participants with varying observations of valid data on depressive symptoms across waves

### Initial growth model of depressive symptoms by perceived social cohesion

Table 2 shows the negative binomial growth model of the number of depressive symptoms regressed on gender; as well as baseline age, PSC and their interaction. The average intercept and slope values, respectively, represent the expected log count of the number of depressive symptoms at baseline, and the rate of change in the expected log count over time—when the combined set of predictors included in each model are all equal to 0.

**Table 2:** Negative binomial growth model of the number of depressive symptoms (2002/2003–2014/2015)

Growth parameters	Initial model^a^			Fully adjusted model^b^		
	*b*	SE	*p*	*b*	SE	*p*
Intercept	− 0.409	0.031	< 0.001	− 0.735	0.043	< 0.001
Intercept regressed on						
Female	0.388	0.022	< 0.001	0.351	0.021	< 0.001
Baseline age (years)^c^	0.023	0.002	< 0.001	0.010	0.002	< 0.001
Medium PSC	0.056	0.033	0.092	0.051	0.030	0.088
Low PSC	0.347	0.033	< 0.001	0.235	0.029	< 0.001
Baseline age × medium PSC	− 0.004	0.003	0.193	− 0.005	0.003	0.097
Baseline age × low PSC	− 0.011	0.003	< 0.001	− 0.011	0.003	< 0.001
Slope^d^	− 0.091	0.048	0.060	− 0.068	0.070	0.335
Slope regressed on						
Female	0.017	0.034	0.621	− 0.007	0.034	0.835
Baseline age (years)	0.015	0.004	< 0.001	0.017	0.004	< 0.001
Medium PSC	0.069	0.048	0.154	0.077	0.047	0.103
Low PSC	0.102	0.046	0.027	0.119	0.045	0.009
Baseline age × medium PSC	0.011	0.005	0.039	0.010	0.005	0.041
Baseline age × low PSC	0.014	0.005	0.003	0.013	0.005	0.007
Intercept variance	0.937	0.023	< 0.001	0.666	0.019	< 0.001
Slope variance	0.557	0.039	< 0.001	0.558	0.039	< 0.001
Intercept–slope covariance	− 0.147	0.026	< 0.001	− 0.127	0.023	< 0.001

^a^Adjusted for the covariates shown in the table, plus white/non-white group and self-reported ever doctor-diagnosis of depression

^b^Adjusted for the covariates shown in the table, plus white/non-white group, self-reported ever doctor-diagnosis of depression, educational qualification, work status, total non-pension wealth, self-rated health, and self-reported limiting long-term illness

^c^Centred at 65 years

^d^As the time scale (years) was divided by 12, the average slope and effect of covariates on the slope describe the total rate of change over the 12-year study period

The intercept value of − 0.409 (SE = 0.031) in the initial model refers to the expected log count of the number of depressive symptoms among men in the high PSC tertile who were aged 65 at baseline. Female counterparts with high PSC at age 65, however, had a higher expected log count of symptoms by 0.388 (SE = 0.022) in comparison to men. These differences were statistically significant, reflecting strong gender differences at baseline. The log count of the number of depressive symptoms increased by 0.023 (SE = 0.002) for each 1 year increase in baseline age from age 65 among men in the reference group (high PSC tertile). The differences in expected log counts of symptoms at baseline were higher by 0.056 (SE = 0.033) and 0.347 (SE = 0.033) among men in the medium and low PSC tertiles, respectively, than men with high PSC at age 65. There was a statistically significant interaction term between baseline age and the low PSC tertile, as the differences in expected log counts at baseline between the high and low PSC categories became marginally smaller with increasing age.

**Fig. 1:**
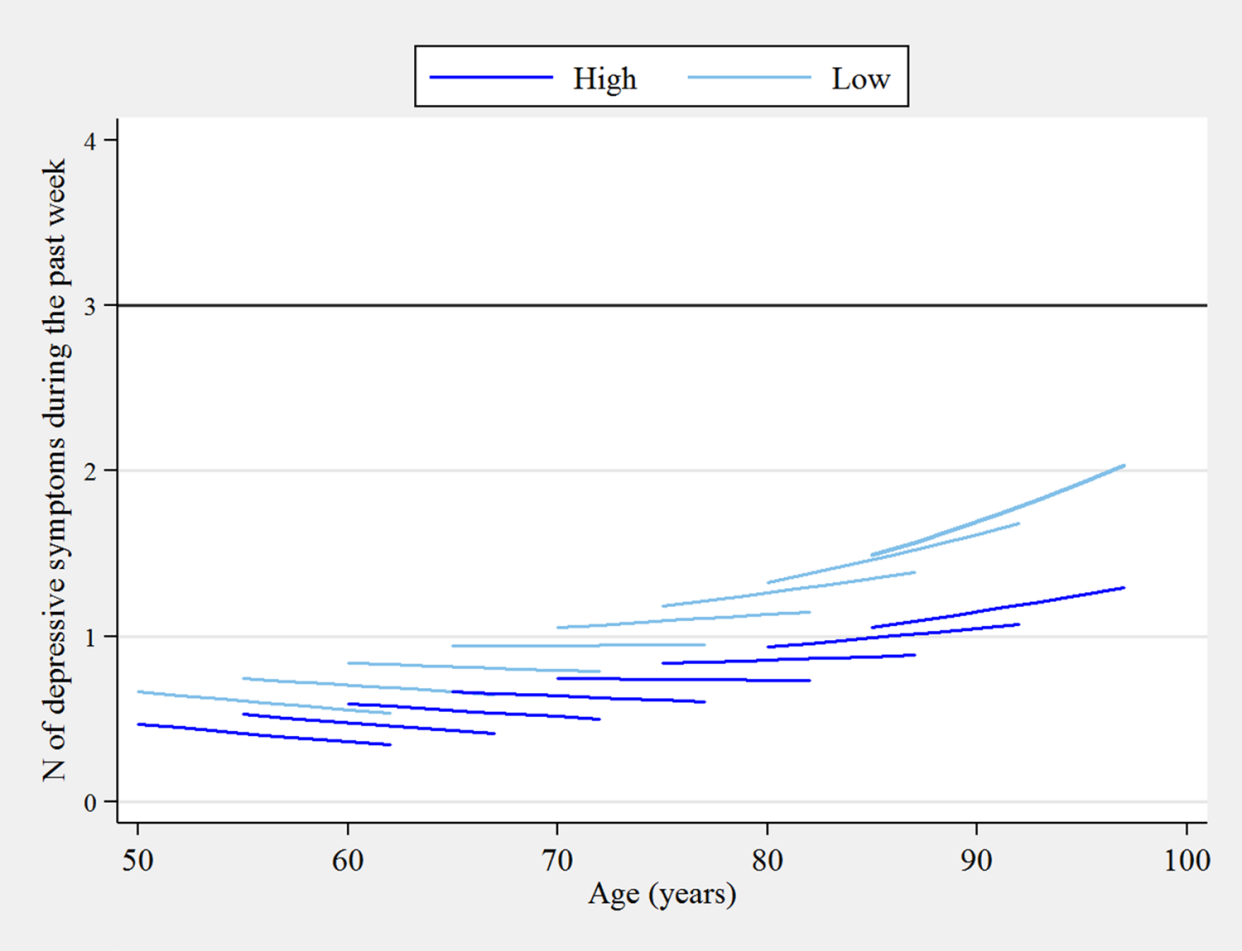
Predicted 12-year ageing vectors of depressive symptoms by high and low perceived social cohesion from the initial growth model (2002/2003–2014/2015) Note: Estimates are adjusted for gender, white/non-white group and self-reported ever doctor-diagnosis of depression

Overall, the expected log count of symptoms decreased by 0.091 (SE = 0.048) over the 12-year follow-up, among men in the high PSC tertile who were aged 65 at baseline. In comparison, the total decline in the expected log count of symptoms for women with high PSC at age 65 was slower by 0.017 (SE = 0.034), but this difference was not statistically significant (*p* = 0.638) over the study period. Combined with the gender difference observed on the intercept, the results indicate that females present a higher number of depressive symptoms at baseline, but the rate of change in symptoms over the 12-year study period is the same as for men. As shown in Fig. 1 however, the rate of change (i.e. slope) was modified by age; at younger ages, the trajectories in depressive symptoms were negative (declining), while at older ages the trajectories became positive (increasing). This is reflected by the significant effect of age in Table 2, showing that the total rate of change in the expected log count was 0.015 (SE = 0.004) higher for every 1-year increase in age from age 65 among men in the high PSC tertile. The estimated rate of change in expected log counts for men in the medium and low PSC tertiles (at age 65) were greater by 0.069 (SE = 0.048) and 0.102 (SE = 0.046), respectively, than men aged 65 with high PSC. Here, the interaction between both PSC tertiles and age were significant on the slope, as the medium and low PSC tertiles exhibited steeper increases in expected log counts of symptoms than the reference category by 0.011 (SE = 0.005) and 0.014 (SE = 0.005), respectively, for each 1-year increase in baseline age from the age of 65.

Figure 1 shows the predicted number of depressive symptoms by high and low PSC tertiles that were calculated and displayed for 5-year cohorts using the initial model estimates. For clarity, trajectories were not plotted for the medium PSC tertile as the main effects for this group were not statistically significant at neither the baseline value nor the total rate of change. At younger ages (until about 60 years) depressive symptoms declined in both high and low PSC tertiles, around the age of 65 the rates of change were generally stable in both groups, and starting from the age of 70 the trajectories increased in both groups and began to increasingly diverge by PSC for each successive 5-year cohort.

### Fully adjusted growth model of depressive symptoms by perceived social cohesion

**Fig. 2:**
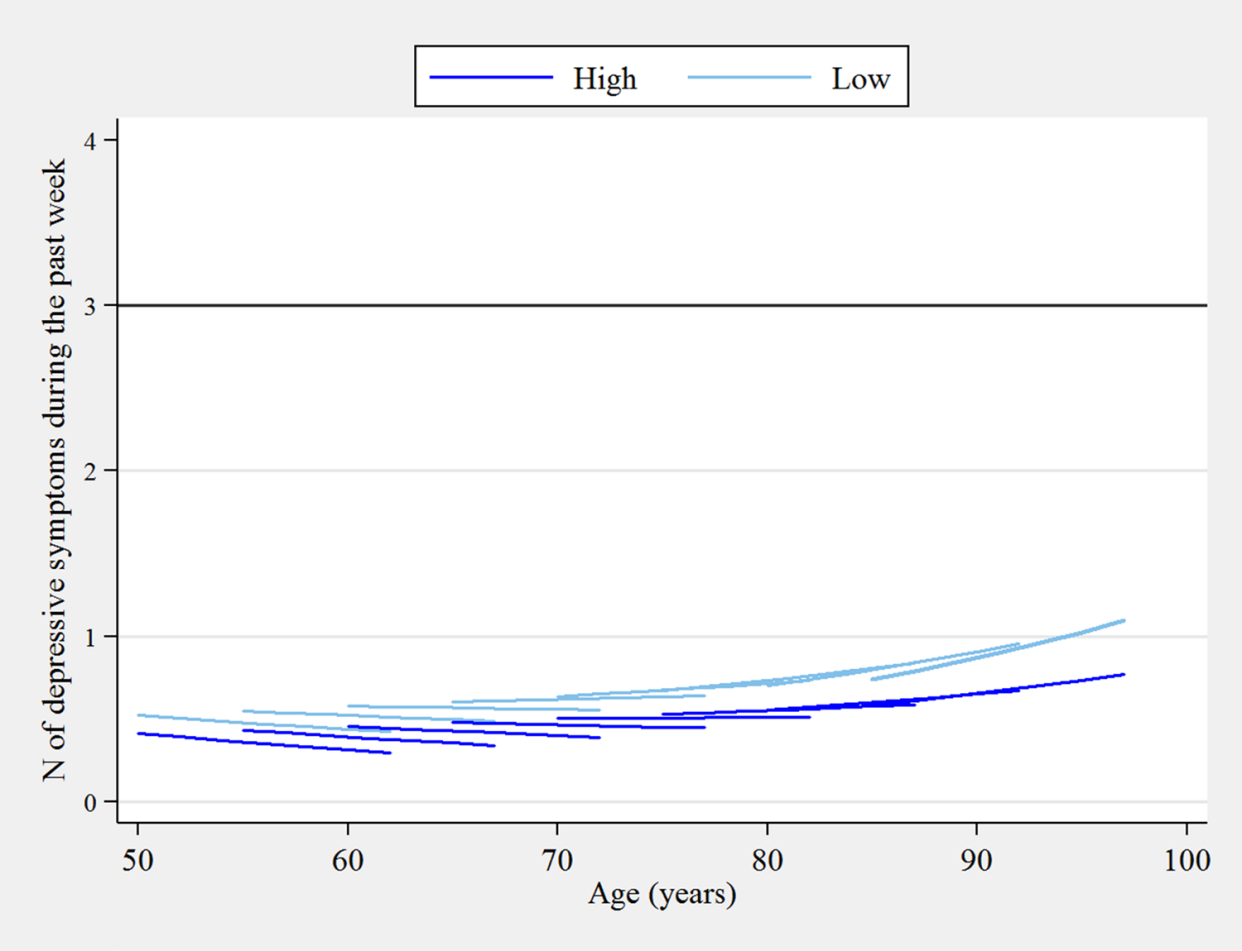
Predicted 12-year ageing vectors of depressive symptoms by high and low perceived social cohesion from the fully-adjusted growth model (2002/2003–2014/2015) Note: Estimates are adjusted for gender, white/non-white group, self-reported ever doctor-diagnosis of depression, educational qualification, work status, total non-pension wealth, self-rated health, and self-reported limiting long-term illness

Further adjustment for socio-economic characteristics and physical health did not change the general pattern of results, as the interaction term effects between age and PSC remained strong. While the adjustment partially attenuated the cross-sectional associations at baseline (i.e. intercept), the 12-year trajectories by age and PSC were not materially changed. This is evident in the depressive symptom trajectories by high and low PSC tertiles that were predicted using the fully adjusted model estimates (Fig. 2). In comparison to the initial model, the predicted number of depressive symptoms decreased for all age cohorts at baseline, and the widening trajectories by high and low PSC were partially attenuated. The life course differences in elevated depressive symptoms by PSC remained, as participants with low PSC in the baseline cohort of age 70 experienced a similar increase in symptoms as those with high PSC in the baseline cohort of age 85.

### Sensitivity analyses

There was no evidence that differences in the number of depressive symptoms at baseline nor the subsequent rates of change between participants with high vs. low PSC were driven by probable depressed cases at baseline who may have reported lower PSC levels in their local area. The main and interaction effects of age and PSC tertiles proved robust after removing probable cases from the sample in the initial and fully adjusted models (Table S2 and Figures S3–S4, supplementary material).

The Kaplan–Meier failure plot illustrated an increased cumulative probability of incident probable depression from high to low PSC tertiles over the 12-year follow-up, which was confirmed by the log-rank test for trend (*p* ≤ 0.001) (Figure S5, supplementary material). An inverse association between lower tertiles of PSC and higher risk of incident probable depression was observed in the initially adjusted model, but the increased rate in the medium PSC tertile was not statistically significant (HR = 1.090, *p* = 0.142). Participants with low PSC, however, were 44% (HR = 1.437, *p* ≤ 0.001) significantly more likely to have incident probable depression over 12 years than high PSC counterparts. The increased HR for the low PSC tertile was partially attenuated, but remained strong at 1.36 (*p* ≤ 0.001) in the fully-adjusted model (Table S6, supplementary material).

## Discussion

This study extends the empirical evidence on the relationship between perceived neighbourhood social cohesion and depressive symptoms among middle-aged and older adults. We found substantial differences in the number of depressive symptoms at baseline and in the rate of change over the 12-year follow-up by PSC. There were significant interaction effects between baseline age and PSC on depressive symptoms in both cross-sectional and prospective analyses. For example, the age-related rise in depressive symptoms among participants with low PSC took place about 15 years earlier in the life course than age-counterparts with high PSC. Moreover, depressive symptom trajectories between participants with low vs. high PSC were different at middle and older ages.

### Limitations and strengths of the study

Several limitations need to be considered. First, the self-reported nature of both the exposure and outcome may lead to a spurious association. A prevailing psychological model of depression is that of cognitive distortions, which postulates, for example, that depressed cases are prone to more negatively evaluate inter-personal relationships and life events as compared to less acute cases and non-depressed persons [1]. While it is plausible that adults with depression may infer low levels of PSC and thus lead to reverse causation, results from the sensitivity analyses do not indicate that our findings are explained by this phenomenon.

Second, as with most epidemiological studies, we relied on self-reported depressive symptoms as we were unable to assess clinically diagnosed depression. While the CES-D scale is a widely validated and commonly used screening scale to monitor depression in the population; it is likely that depressive symptoms, especially for low-scoring participants, reflect less severe affect states or transient symptoms. Although we could not test whether PSC was associated with clinical cases of depression; we did, however, assess whether associations were found with the onset of clinically-relevant depressive symptoms. These additional analyses confirmed that PSC was not only significantly associated with ageing trajectories of depressive symptoms, but also with the incidence of clinically relevant symptomatology over the study period. In other words, the life course differences in the age-related rise in depressive symptoms between adults with low vs. high PSC mirrored clinically significant differences in the risk of probable depression.

Third, similar to most longitudinal studies, there was substantial attrition of the cohort over follow-up, raising questions about the representativeness of the study sample for the general population. The mean number of depressive symptoms over the 12-year follow-up was well below three symptoms [26], an established cut-off for the abridged CES-D 8 for possible depression, for all age cohorts. This accords with previous research of long-term trajectories (5 years or more) that found that most participants consistently reported few to no depressive symptoms over follow-up [33]. Similarly, the population distribution of PSC scores was negatively skewed, with fewer participants reporting negatively on their local area at baseline. It is likely that our sample had fewer depressive symptoms and lived in neighbourhoods with higher PSC than the general English population. Bias due to non-response, attrition and death at older ages were dealt with by employing population weighting for participant non-response and multiple imputation for item non-response, simultaneously with full information maximum likelihood for attrition and death. Nevertheless, the low prevalence of depressive symptoms and high levels of PSC may lead to an underestimation of the true difference in depressive symptom trajectories by PSC.

On the other hand, our study also has considerable strengths. The original sampling frame of the ELSA study was population-based, and participant data were collected using a robust study protocol and subject to rigorous quality control. ELSA is widely considered as a high-quality ageing study with as complete follow-up as feasibly possible, and the analytic sample size provided large statistical power over a 12-year period. We used a combination of techniques to deal with the cohort non-response and attrition.

### Interpretation of results

Most of the existing evidence between neighbourhood social cohesion and depressive symptoms in later life is based on cross-sectional studies [12, 13, 17, 18]. The cross-sectional association at baseline reported in our findings are consistent with this overall pattern from this literature. By contrast, relatively few studies investigated whether cognitive/perceptual social capital influences the rate of change in depressive symptomatology in later life, with inconsistent results [14, 15]. In a study of older US adults, individual-level and neighbourhood-level markers of perceived social cohesion were both linked with fewer depressive symptoms at baseline, but effects on the growth rate in symptoms were not statistically significant for either measure over the 10-year follow-up [14]. Among older Korean women, however, perceived trust and reciprocity among neighbours were independently linked with fewer depressive symptoms at baseline and a faster rate of decline over 7 years [15].

PSC is a social process among individuals nested within neighbourhoods. Therefore, it is important to tease apart the individual and neighbourhood dimensions of the measure to rigorously assess its role on depressive symptoms. A multi-level latent growth model recommended by the literature [9] was unfortunately not possible in this study, due to the lack of access to data on area-level characteristics of study participants. As we were unable to derive a neighbourhood-level marker of PSC using area-level data in addition to the individual-level measure employed in the present paper, potential information bias remains an issue and this may partly explain the differences between our findings and previous studies.

Several pathways have been proposed by which neighbourhood social processes may influence depression, including the level of neighbourhood-based stress placed on residents, the creation of strong and protective social networks; personal resiliency to stress and negative affect; and personal level of agency and control in their area of residence [8]. The literature on older adults suggest the association of depression with social cohesion is partly mediated by a personal sense of control and friendship quality [16] as well as frequency of going outside one’s home [11]. As each of these factors are often curtailed amidst the ageing process, it is plausible that the effects of low PSC on depression are greater as adults reach old age. Our report of widening depressive symptom trajectories by PSC with age may be accurate for several reasons. First, declines in physical and cognitive health decreases older adults’ capacity to handle environmental stressors. Second, residents may feel more defenceless in neighbourhoods with certain adverse qualities as they age. Third, loss of loved ones and children moving out of the local area reduce social support and may obligate the elderly to rely on community resources instead of individual resources. Lastly, decreased mobility disposes older adults to be more confined to their residential area [17–21].

A life course perspective on depression suggests that it is not merely a clinical disorder, but an affect state that spans from transient mood variations or situational depressive reaction to difficult life events, to more serious and persistent disabling disorders [34]. There is a long-standing and nuanced debate on the case definition of the disorder for older adults, as proposed sub-types include: major depression; minor, subsyndromal or subthreshold depression; depression without sadness; vascular depression and depression of Alzheimer’s disease; among others [1]. As the CES-D scale measures the presence of clinically relevant symptoms that characterise a wide range of depressive states [24, 27], the present paper analysed total CES-D 8 scores to track subtle changes in depressive symptom severity through mid and later life, and to provide a picture of depression risk below the clinical iceberg.

As many life changes experienced from middle to older ages may influence this continuum of risk of depression, it is important to better understand the mechanisms behind widening depressive symptom trajectories by PSC as people age. Future research is needed on how best to reduce the unequal onset of risk by psycho-social processes that may be ameliorated by individual- and community-level social policies. Our findings suggest that mental health policy interventions should target adults early in the ageing process, and prior to the period where PSC inequalities become exacerbated.

## Electronic supplementary material

Below is the link to the electronic supplementary material.

Supplementary material 1 (DOCX 237 KB)
