# Supplementary material for: Perceived neighbourhood social cohesion and depressive symptom trajectories in older adults: a 12-year prospective cohort study
Source: Soc Psychiatry Psychiatr Epidemiol. 2018 Jun 19;53(10):1081–90. doi: 10.1007/s00127-018-1548-4 (PMC6182502; doi:10.1007/s00127-018-1548-4)
Supplement: Supplementary file 1 — Supplementary material 1 (DOCX 237 KB) [file 127_2018_1548_MOESM1_ESM.docx]

**Title:** Perceived neighbourhood social cohesion and depressive symptom trajectories in older adults: A twelve-year prospective cohort study

**Journal:** Social Psychiatry and Psychiatric Epidemiology

**Authors:** Milagros Ruiz, PhD, MSc^1^, Shaun Scholes, PhD, MSc^1^, Martin Bobak, PhD, MD, MSc^1^

**Affiliation and e-mail address of corresponding author:** ^1^Research Department of Epidemiology and Public Health, University College London, 1-19 Torrington Place, London, WC1E 6BT, United Kingdom, m.a.ruiz@ucl.ac.uk

**Supplementary Material**

S1 Study characteristics of participants with ≤ 2 depressive symptoms at baseline in the longitudinal analytic sample 2

S2 Poisson growth model of the number of depressive symptoms for participants with ≤ 2 symptoms at baseline (2002/3 – 2014/5) 3

S3 Predicted 12-year ageing vectors of depressive symptoms by high and low perceived social cohesion from the initial growth model for participants with ≤ 2 symptoms at baseline (2002/3 – 2014/5) 4

S4 Predicted 12-year ageing vectors of depressive symptoms by high and low perceived social cohesion from the fully-adjusted growth model for participants with ≤ 2 symptoms at baseline (2002/3 – 2014/5) 5

S5 Kaplan-Meier failure plot of incident probable depression (3 ≥ depressive symptoms) by tertiles of perceived social cohesion for participants with ≤ 2 symptoms at baseline (2002/3 – 2014/5) 6

S6 Discrete time proportional hazard model of incident probable depression (3 ≥ depressive symptoms) by tertiles of perceived social cohesion for participants with ≤ 2 symptoms at baseline (2002/3 – 2014/5) 6

## S1 Study characteristics of participants with ≤ 2 depressive symptoms at baseline in the longitudinal analytic sample

| **Study data^a^** | | |
| --- | --- | --- |
| *Longitudinal measures* |  |  |
| Number of depressive symptoms during the past week (0-8) | **Mean** | **N** |
| Wave 1 (2002/3) | 0.6 | 8,269 |
| Wave 2 (2004/5) | 1.1 | 6,516 |
| Wave 3 (2006/7) | 1.0 | 5,590 |
| Wave 4 (2008/9) | 1.0 | 4,927 |
| Wave 5 (2010/1) | 1.1 | 4,594 |
| Wave 6 (2012/3) | 1.0 | 4,201 |
| Wave 7 (2014/5) | 1.0 | 3,637 |
| Probable depression (3 ≥ symptoms during the past week) | **%** | **N** |
| Wave 1 (2002/3) | 0.0 | 8,269 |
| Wave 2 (2004/5) | 14.0 | 6,516 |
| Wave 3 (2006/7) | 13.1 | 5,590 |
| Wave 4 (2008/9) | 14.3 | 4,927 |
| Wave 5 (2010/1) | 15.4 | 4,594 |
| Wave 6 (2012/3) | 13.4 | 4,201 |
| Wave 7 (2014/5) | 13.8 | 3,637 |
| *Baseline measures* | **Mean or %** | **N** |
| Perceived social cohesion score (0-24) |  |  |
| High (22 – 24) | 33.1 | 2,737 |
| Medium (18 – 21) | 34.0 | 2,811 |
| Low (0 – 17) | 28.2 | 2,721 |
| Baseline age | 64.6 | 8,269 |
| Female | 51.0 | 4,217 |
| Non-white | 2.2 | 182 |
| Self-reported ever doctor-diagnosis of depression | 3.3 | 273 |
| No educational qualification | 38.7 | 3,200 |
| Work status |  |  |
| Employed | 36.9 | 3,051 |
| Retired | 49.5 | 4,093 |
| Economically inactive | 4.2 | 347 |
| Permanently sick/disabled | 9.4 | 777 |
| Total non-pension wealth |  |  |
| 1 (Richest) | 15.7 | 1,298 |
| 2 | 18.4 | 1,521 |
| 3 | 20.7 | 1,712 |
| 4 | 22.1 | 1,827 |
| 5 | 23.1 | 1,910 |
| Self-rated health |  |  |
| Very good | 23.9 | 1,976 |
| Good | 38.3 | 3,167 |
| Fair | 25.9 | 2,142 |
| Poor | 11.9 | 984 |
| Self-reported limiting long-term illness | 26.4 | 2,183 |

a: The estimates are averaged over the multiply imputed data sets, and corrected for the study’s non-response at wave 1. The longitudinal analytic sample (n=8,269) includes participants with varying observations of valid data on depressive symptoms across waves.

## S2 Poisson growth model of the number of depressive symptoms for participants with ≤ 2 symptoms at baseline (2002/3 – 2014/5)

|  | **Initial model^a^** | | | **Fully-adjusted model^b^** | | |
| --- | --- | --- | --- | --- | --- | --- |
| **Growth parameters** | **b** | **SE** | **P** | **b** | **SE** | **P** |
| Intercept | -0.814 | 0.032 | <0.001 | -0.977 | 0.048 | <0.001 |
| Intercept regressed on | | | | | | |
| Female | 0.336 | 0.025 | <0.001 | 0.340 | 0.025 | <0.001 |
| Baseline age (years)^d^ | 0.016 | 0.002 | <0.001 | 0.009 | 0.002 | <0.001 |
| Medium PSC | 0.096 | 0.035 | 0.006 | 0.094 | 0.035 | 0.007 |
| Low PSC | 0.244 | 0.034 | <0.001 | 0.195 | 0.033 | <0.001 |
| Baseline age x medium PSC | -0.004 | 0.003 | 0.259 | -0.003 | 0.003 | 0.294 |
| Baseline age x low PSC | -0.007 | 0.003 | 0.016 | -0.008 | 0.003 | 0.010 |
| Slope^c^ | 0.274 | 0.058 | <0.001 | 0.453 | 0.019 | <0.001 |
| Slope regressed on | | | | | | |
| Female | 0.086 | 0.041 | 0.036 | 0.043 | 0.042 | 0.312 |
| Baseline age (years) | 0.023 | 0.004 | <0.001 | 0.021 | 0.004 | <0.001 |
| Medium PSC | 0.002 | 0.056 | 0.968 | 0.010 | 0.056 | 0.858 |
| Low PSC | 0.122 | 0.056 | 0.030 | 0.121 | 0.057 | 0.032 |
| Baseline age x medium PSC | 0.012 | 0.006 | 0.045 | 0.011 | 0.006 | 0.067 |
| Baseline age x low PSC | 0.017 | 0.006 | 0.003 | 0.016 | 0.006 | 0.007 |
| Intercept variance | 0.545 | 0.020 | <0.001 | 0.788 | 0.038 | <0.001 |
| Slope variance | 0.423 | 0.038 | <0.001 | 0.420 | 0.038 | <0.001 |
| Intercept – Slope covariance | 0.156 | 0.023 | <0.001 | 0.125 | 0.021 | <0.001 |

a*:* Adjusted for the covariates shown in the table, plus white/non-white group and self-reported ever doctor-diagnosis of depression.

b: Adjusted for the covariates shown in the table, plus white/non-white group, self-reported ever doctor-diagnosis of depression, educational qualification, work status, total non-pension wealth, self-rated health, and self-reported limiting long-term illness.

c: As the time scale (years) was divided by 12, the average slope and effect of covariates on the slope describe the total rate of change over the twelve-year study period.

d: Centred at 65 years.

## S3 Predicted 12-year ageing vectors of depressive symptoms by high and low perceived social cohesion from the initial growth model for participants with ≤ 2 symptoms at baseline (2002/3 – 2014/5)


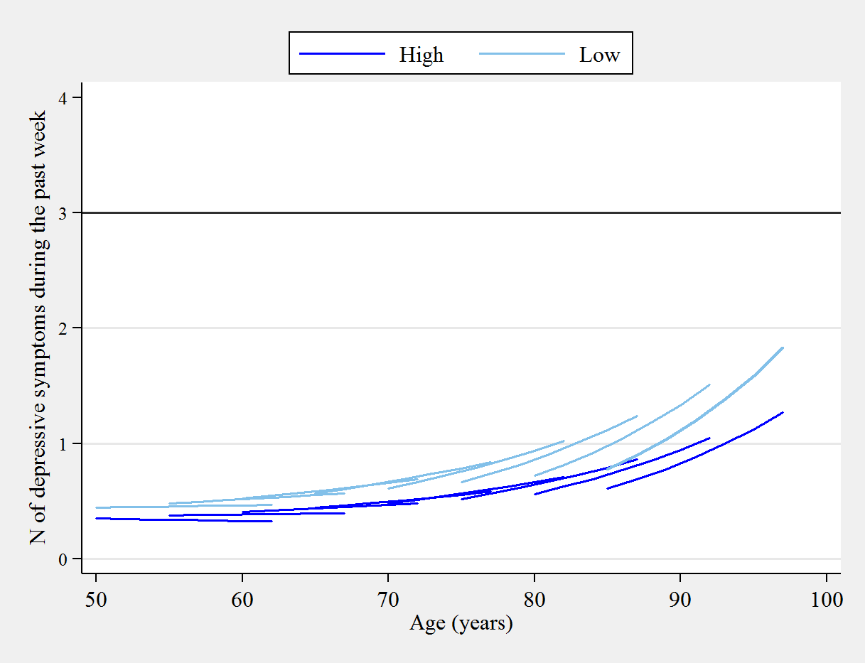


*Note:* Estimates are adjusted for gender, white/non-white group and self-reported ever doctor-diagnosis of depression.

## S4 Predicted 12-year ageing vectors of depressive symptoms by high and low perceived social cohesion from the fully-adjusted growth model for participants with ≤ 2 symptoms at baseline (2002/3 – 2014/5)


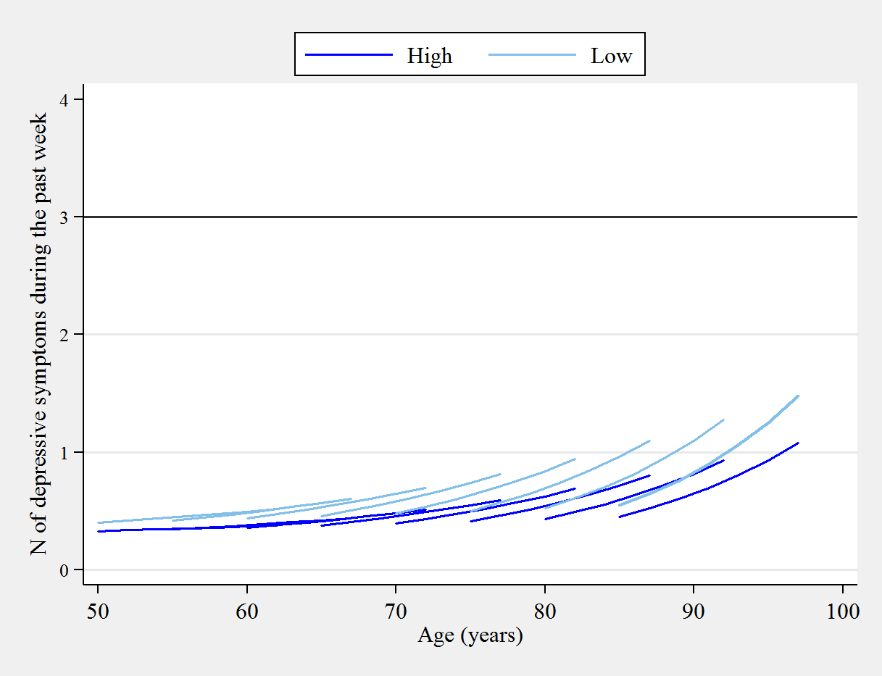


*Note:* Estimates are adjusted for gender, white/non-white group, self-reported ever doctor-diagnosis of depression, educational qualification, work status, total non-pension wealth, self-rated health, and self-reported limiting long-term illness.

## S5 Kaplan-Meier failure plot of incident probable depression (3 ≥ depressive symptoms) by tertiles of perceived social cohesion for participants with ≤ 2 symptoms at baseline (2002/3 – 2014/5)


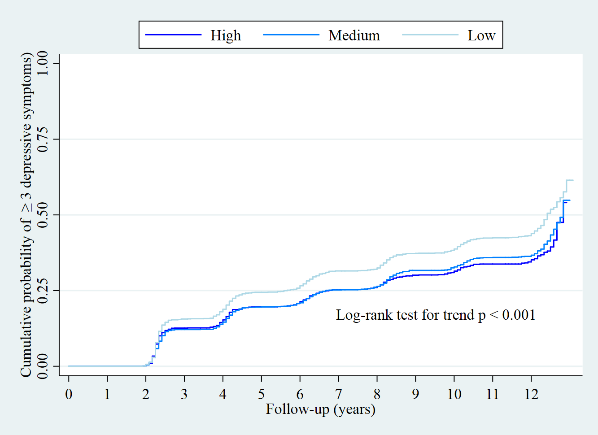


*Note:* Follow-up time (months) was rescaled to years for ease of interpretation.

## S6 Discrete time proportional hazard model of incident probable depression (3 ≥ depressive symptoms) by tertiles of perceived social cohesion for participants with ≤ 2 symptoms at baseline (2002/3 – 2014/5)

|  | **Initial model^a^** | | | **Fully-adjusted model^b^** | | |
| --- | --- | --- | --- | --- | --- | --- |
| **Estimates** | **HR** | **SE** | **P** | **HR** | **SE** | **P** |
| Female | 1.529 | 0.069 | <0.001 | 1.477 | 0.069 | <0.001 |
| Baseline age (years)^c^ | 1.042 | 0.003 | <0.001 | 1.036 | 0.003 | <0.001 |
| Perceived social cohesion |  |  |  |  |  |  |
| High | 1.000 | - | - | 1.000 | - | - |
| Medium | 1.090 | 0.064 | 0.142 | 1.089 | 0.063 | 0.138 |
| Low | 1.437 | 0.082 | <0.001 | 1.356 | 0.076 | <0.001 |

a*:* Adjusted for the covariates shown in the table, plus white/non-white group and self-reported ever doctor-diagnosis of depression.

b: Adjusted for the covariates shown in the table, plus white/non-white group, self-reported ever doctor-diagnosis of depression, educational qualification, work status, total non-pension wealth, self-rated health, and self-reported limiting long-term illness.

c: Centred at 65 years.
